# Transcriptomics analysis of long non-coding RNAs in smooth muscle cells from patients with peripheral artery disease and diabetes mellitus

**DOI:** 10.1038/s41598-024-59164-7

**Published:** 2024-04-14

**Authors:** Yankey Yundung, Shafeeq Mohammed, Francesco Paneni, Benedikt Reutersberg, Fabian Rössler, Alexander Zimmermann, Jaroslav Pelisek

**Affiliations:** 1 Experimental Vascular Surgery/Department of Vascular Surgery, University Hospital Zurich/University of Zurich, Schlieren, Switzerland; 2 Department of Cardiology/Center for Translational and Experimental Cardiology (CTEC), University Hospital Zurich/University of Zurich, Schlieren, Switzerland; 3 Department of Surgery and Transplantation, University Hospital Zurich, Zürich, Switzerland

**Keywords:** Molecular medicine, Cardiovascular biology

## Abstract

Diabetes mellitus (DM) is a significant risk factor for peripheral arterial disease (PAD), and PAD is an independent predictor of cardiovascular disorders (CVDs). Growing evidence suggests that long non-coding RNAs (lncRNAs) significantly contribute to disease development and underlying complications, particularly affecting smooth muscle cells (SMCs). So far, no study has focused on transcriptome analysis of lncRNAs in PAD patients with and without DM. Tissue samples were obtained from our Vascular Biobank. Due to the sample’s heterogeneity, expression analysis of lncRNAs in whole tissue detected only ACTA2-AS1 with a 4.9-fold increase in PAD patients with DM. In contrast, transcriptomics of SMCs revealed 28 lncRNAs significantly differentially expressed between PAD with and without DM (FDR < 0.1). Sixteen lncRNAs were of unknown function, six were described in cancer, one connected with macrophages polarisation, and four were associated with CVDs, mainly with SMC function and phenotypic switch (NEAT1, MIR100HG, HIF1A-AS3, and MRI29B2CHG). The enrichment analysis detected additional lncRNAs H19, CARMN, FTX, and MEG3 linked with DM. Our study revealed several lncRNAs in diabetic PAD patients associated with the physiological function of SMCs. These lncRNAs might serve as potential therapeutic targets to improve the function of SMCs within the diseased tissue and, thus, the clinical outcome.

## Introduction

Peripheral arterial disease (PAD), defined as the narrowing of the peripheral arteries, is primarily caused by atherosclerotic changes within the vessel wall, particularly in the lower extremities. Diabetes mellitus (DM) is a significant risk factor for PAD, and PAD is an independent predictor of cardiovascular and cerebrovascular ischemic events, affecting both the quality and expectancy of life^1–3^. In diabetic patients, atherosclerotic lesions occur earlier with rapid progression and are frequently asymptomatic, thus bearing a high risk of unexpected cardiac and cerebral complications^2,3^. Multiple metabolic aberrations, such as overproduction of advanced glycation end-products (AGEs), increased oxidative stress, reactive oxygen species (ROS), enhanced inflammation, and dyslipidaemia, have been shown to aggravate PAD in patients with DM^3–5^. The metabolic changes strongly affect the biological function of endothelial cells (ECs) and upregulate the expression of many inflammatory factors^6–8^. These cytokines, in turn, promote atherosclerosis as well as apoptosis of ECs. Increased inflammation, as well as oxidative stress, facilitate the development of atherosclerotic lesions within the arterial wall. In PAD patients, atherosclerosis increases the risk of thrombosis and lower extremity ulceration^1,2^. Furthermore, individuals with PAD suffering from DM are more susceptible to plaque rupture^9,10^. In addition, hyperglycaemia in diabetic patients induces the production of ROS and AGEs, leading to vascular damage and diminished bioavailability of nitric oxide (NO)^3,6,11,12^. NO and prostacyclin PGI2 are the essential vasoactive factors affecting the underlying smooth muscle cells (SMCs)^3,13–16^. The impaired ECs, together with other metabolic aberrations due to DM, impair the physiological function of SMCs by changing their phenotype in a proatherogenic manner, leading increasingly also to apoptosis^17,18^.

Growing evidence suggests that particularly long non-coding RNAs (lncRNAs), participating in many biological processes such as transcriptional regulation of oxidative stress, inflammation, atherosclerosis, as well as insulin sensitivity, may also affect PAD development, especially in patients suffering from DM^19,20^. Furthermore, many lncRNAs have already been described to significantly affect the phenotype and the physiological behaviour of vascular cells in DM, as well as the crosstalk between ECs and SMCs^21,22^. Thus, dysregulation of lncRNAs in DM patients suffering from PAD may significantly affect the biological function mainly observed in SMCs and thus aggravate the clinical outcome for these patients. Combining clinical data with omics analyses and bioinformatics is a useful strategy for improving diagnostic precision and pursuing personalised medicine in the treatment of CVDs. Particularly, bioinformatics analysis of big data and the use of artificial intelligence may help to discover novel useful targets or biomarkers with the potential for more accurate diagnosis and therapy^23–25^.

Interestingly, no study has so far focused on the analysis of lncRNAs in diabetic patients with PAD using RNA sequencing of the corresponding tissue. Performing an extended PubMed research, we found no corresponding relevant work dealing with PAD or DM patients and lncRNAs, particularly in humans. Therefore, having the advantage of possessing such tissue samples in our vascular biobank, we performed a detailed transcriptome analysis of PAD patients with and without DM, focusing particularly on lncRNAs and SMCs.

## Results

### Patient characteristics

The clinical characteristics of the selected study patients included in the transcriptome analysis of lncRNA, associated diseases, and corresponding medication are summarised in Table 1. No significant differences were observed between the study groups of PAD patients with and without DM with regard to age, gender or any other collected clinical data. The mean age of PAD patients with and without DM was 74.9 ± 9.2 and 71.9 ± 11.2 years, 52.9% and 40.0% were of the male sex. Most of the patients suffered from chronic kidney disease (82.4% and 80.0%). Furthermore, 58.8% and 65.0 of the study participants received aspirin or clopidogrel, 64.8% and 40.0% beta-blockers and/or ACE inhibitors, and 64.7 and 75.0% were on statins. The only divergence was observed for diuretic intake, with 52.5% of individuals suffering from DM compared to 15.0% of patients without DM (*P* = 0.038).

**Table 1:** Study patients' clinical data.

	DMplus	DMminus	*P* value
	(n = 17)	(n = 20)	
Age (years)	74.9 ± 9.2	71.9 ± 11.2	0.240
Sex (male)	52.9%	40.0%	0.340
Hypertension	94.1%	80.0%	0.495
Hyperlipidaemia	70.6%	60.0%	0.495
Smoking	41.2%	35.0%	1.000
Chronic kidney disease	82.4%	80.0%	1.000
Cardiovascular disease	47.1%	25.0%	0.313
Stroke	5.9%	10.0%	1.000
Aspirin / Clopidogrel	58.8%	65.0%	0.508
Beta-blocker	64.8%	40.0%	0.194
ACE inhibitors	41.2%	45.0%	0.743
Statins	64.7%	75.0%	0.728
Diuretics	52.9%	15.0%	0.038

### Pathomorphological analysis

**Figure 1:**
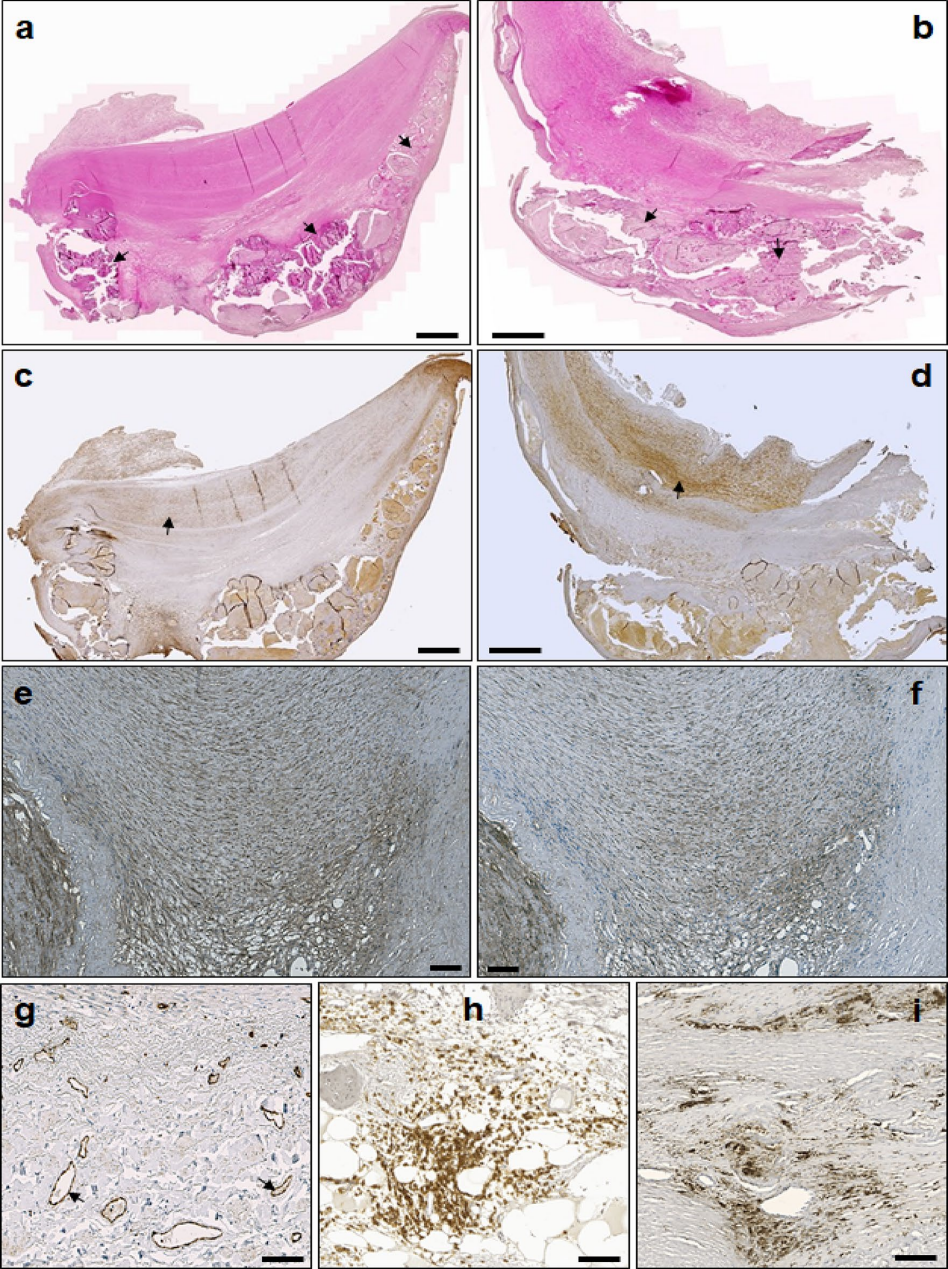
Selected histological examples of the tissue samples from PAD patients with and without DM. (**a**) and (**b**) Haemalaun-Eosin staining of PAD tissue with and without DM. Most samples were highly atherosclerotic and heavily calcified (arrows). (**c**) and (**d**) Smooth muscle actin staining. (**e**) and (**f**): Example of MYH10 (synthetic phenotype of SMCs) and MYH11 (contractile phenotype of SMCs) staining. High overlapping was observed. (**g**) Example of ECs staining using CD31. (**h**) Example of leukocyte staining using CD45. (**i**) Example of macrophage staining using CD68. Scale bars: 1 mm (**a**–**d**), 100 µm (**e**–**i**).

At first, we performed histological and immunohistochemical (IHC) staining of the FFPE samples from the diseased iliac artery (n = 79) to obtain an insight into the pathomorphology of the tissue of our study patients. The specimen characterisation revealed highly heterogeneous pathophysiological features (Fig. 1).

Most samples had extended atherosclerotic lesions, inflammation, calcification, and were highly vascularised. Interestingly, staining with MYH10 and MYH11 to distinguish between the synthetic and contractile phenotype of SMCs showed high overlapping characteristics and no clear separation between these two morphologic features was observed (Fig. 1e and f). In order to reduce the broad heterogeneity of our study tissue, samples with a great extent of calcification, inflammation, and a low number of cells, which markedly differed from the average, were excluded. Consequently, from the 79 histologically characterised patients, 37 were finally included in the transcriptomics analysis (DMplus: n = 17, DMminus: n = 20). The semi-quantitative pathomorphological analysis of the included study samples revealed no significant differences between the study groups (Table 2).

**Table 2:** Histological characterisation* of the tissue samples.

	DMplus	DMminus	*P* value
	(n = 17)	(n = 20)	
Infiltrates	20.3%	25.5%	0.150
SMCs	43.4%	50.8%	0.822
Neovessel	27.3%	33.3%	0.233
Thrombus (extent)	29.3%	19.8%	0.111
Atherosclerosis (extent)	19.3%	18.3%	0.893
Calcification / extent	11.6%	15.8%	0.518

*Using HE and EvG staining, the individual pathological features of the tissue samples were evaluated semi-quantitatively. 100% corresponds to the highest observed occurrence of the individual features.

### Differential expression analysis of lncRNA in the whole PAD tissue samples

**Figure 2:**
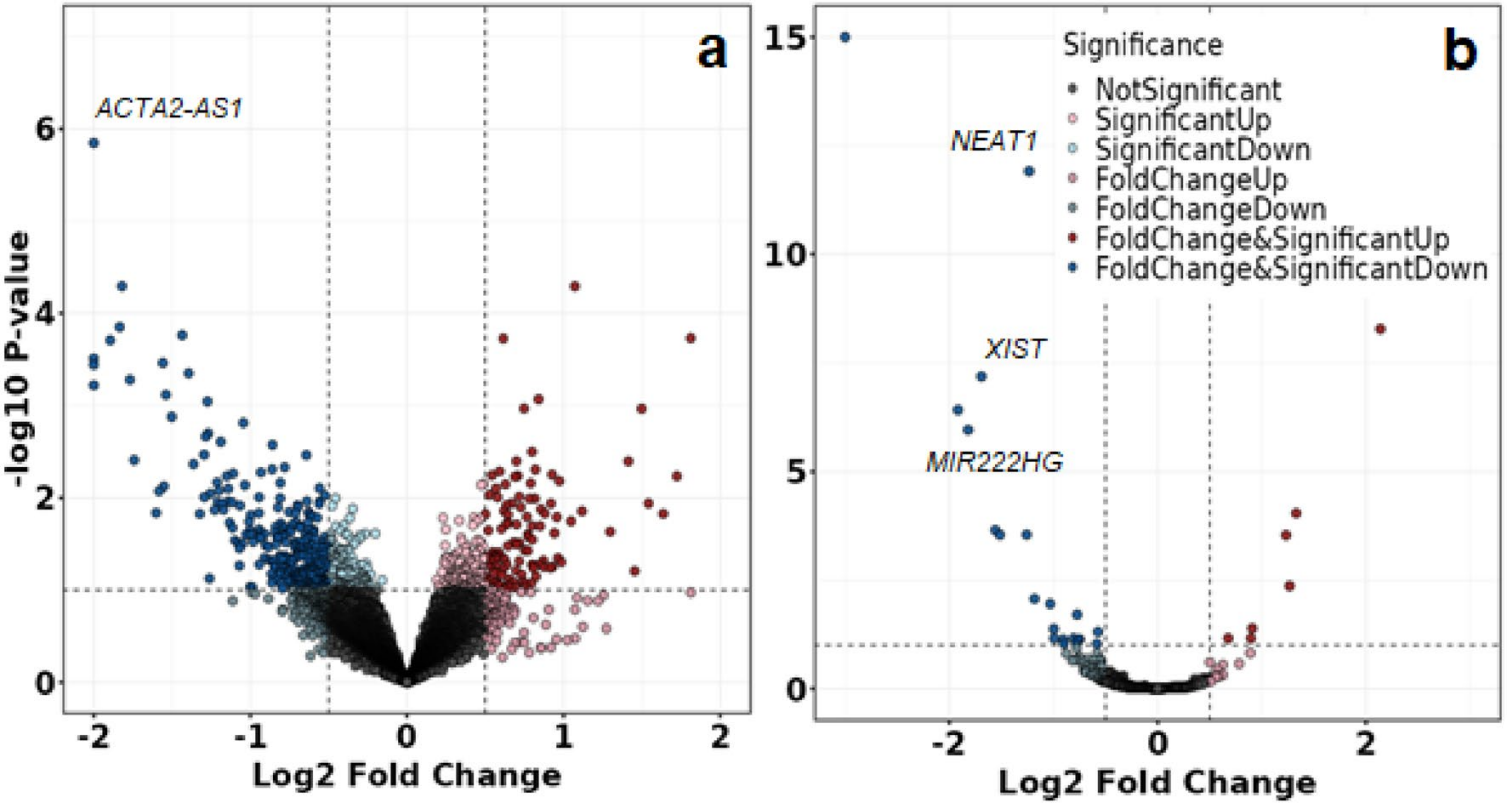
Volcano plot comparing differentially expressed lncRNAs in the whole tissue (**a**) and in SMCs (**b**) from PAD patients with and without DM (DMplus–over–DMminus). The plot shows − log10 transformed FDR as a function of the difference between study groups. The broken lines show 0.5 log2 fold change. Statistically significant differences: FDR < 0.1.

First, we analysed the RNA from the whole tissue samples, focusing on lncRNAs. The volcano plot, comparing PAD patients with and without DM, is shown in Fig. 2. In total, 13,491 lncRNAs were detected, with 6672 above the threshold of 10 counts per million. Nevertheless, only one lncRNA, *ACTA2-AS1*, demonstrated significantly differential expression between the study groups (4.9-fold increase in DMplus samples, FDR = 0.031, P = 2.7E−06) (Fig. 2a, Table 3).

**Table 3:** LncRNAs with significant expression differences between study groups (FDR *P*-value < 0.1).

Gene symbol	Fold change	*P*-value	FDR *P*-value	Gene function (if described)	Literature
DMplus versus DMminus (whole tissue)					
***ACTA2-AS1***	4.90	2.7E−06	0.031	phenotypic switch SMCs, proliferation and migration of cancer cells	^26–28^
DMplus versus DMminus (SMCs)					
*ENSG00000289474*	− 12.21	7.7E−27	5.8E−24	*Novel transcript*	–
***NEAT1***	− 2.35	3.2E−15	1.2E−12	SMC phenotype, proliferation, atherosclerosis, aneurysm	^41,44,46,52–56^
*ENSG00000288156*	− 0.23	2.1E−11	5.3E−09	*Novel transcript*	–
***XIST***	− 3.23	3.4E−10	6.5E−08	SMC proliferation, migration, apoptosis	^51,57,58^
*ENSG00000289901*	− 3.73	2.5E−09	6.5E−08	*Novel transcript*	–
***MIR222HG***	− 3.53	8.6E−09	1.1E−06	Macrophages polarisation	^37,38^
*LINC01220*	− 0.40	8.3E−07	9.1E−05	Cancer	^33^
*CYTOR*	− 2.95	2.3E−06	2.2E−04	Cell proliferation and migration in cancer	^35^
*ENSG00000288794*	− 2.39	3.4E−06	2.8E−04	*Novel transcript*	–
*ENSG00000288928*	− 2.86	3.8E−06	2.9E−04	*Novel transcript*	–
*LINC00910*	2.35	4.2E−06	2.9E−04	Colorectal and breast cancer	^34^
*ENSG00000291174*	2.41	6.8E−05	0.0043	*Novel transcript*	–
*LINC00511*	− 2.27	1.5E−04	0.0085	Cell proliferation in cancer	^32^
*ENSG00000267520*	− 2.04	2.1E−04	0.0111	*Novel transcript*	–
*MIR23AHG*	− 1.71	3.9E−04	0.0196	*Function unknown*	–
*RUFY1-AS1*	1.88	8.6E−04	0.0409	*Function unknown*	–
*ENSG00000271959*	− 2.00	9.4E−04	0.0422	*Novel transcript*	–
*ENSG00000248994*	− 1.49	0.0012	0.0492	*Novel transcript*	–
***MIR100HG***	− 1.99	0.0019	0.0692	Regulator of cell proliferation, cardiomyopathy	^48^
*PURPL*	1.60	0.0019	0.0692	Regulator of cell proliferation in cancer	^30^
*ENSG00000281195*	− 1.75	0.0020	0.0692	*Novel transcript*	–
*MZF1-AS1*	1.86	0.0022	0.0693	Cancer	^31^
***HIF1A-AS3***	− 1.88	0.0023	0.0728	SMC phenotype	^47,50,59,60^
*ENSG00000290021*	− 1.68	0.0023	0.0729	*Novel transcript*	–
*ENSG00000279175*	− 1.69	0.0024	0.0740	*Novel transcript*	–
***MIR29B2CHG***	− 1.87	0.0032	0.0924	Heart failure	^42^
*ENSG00000289404*	− 1.50	0.0033	0.0925	*Novel transcript*	–
*ENSG00000269940*	− 1.72	0.0035	0.0940	*Novel transcript*	–

**Figure 3:**
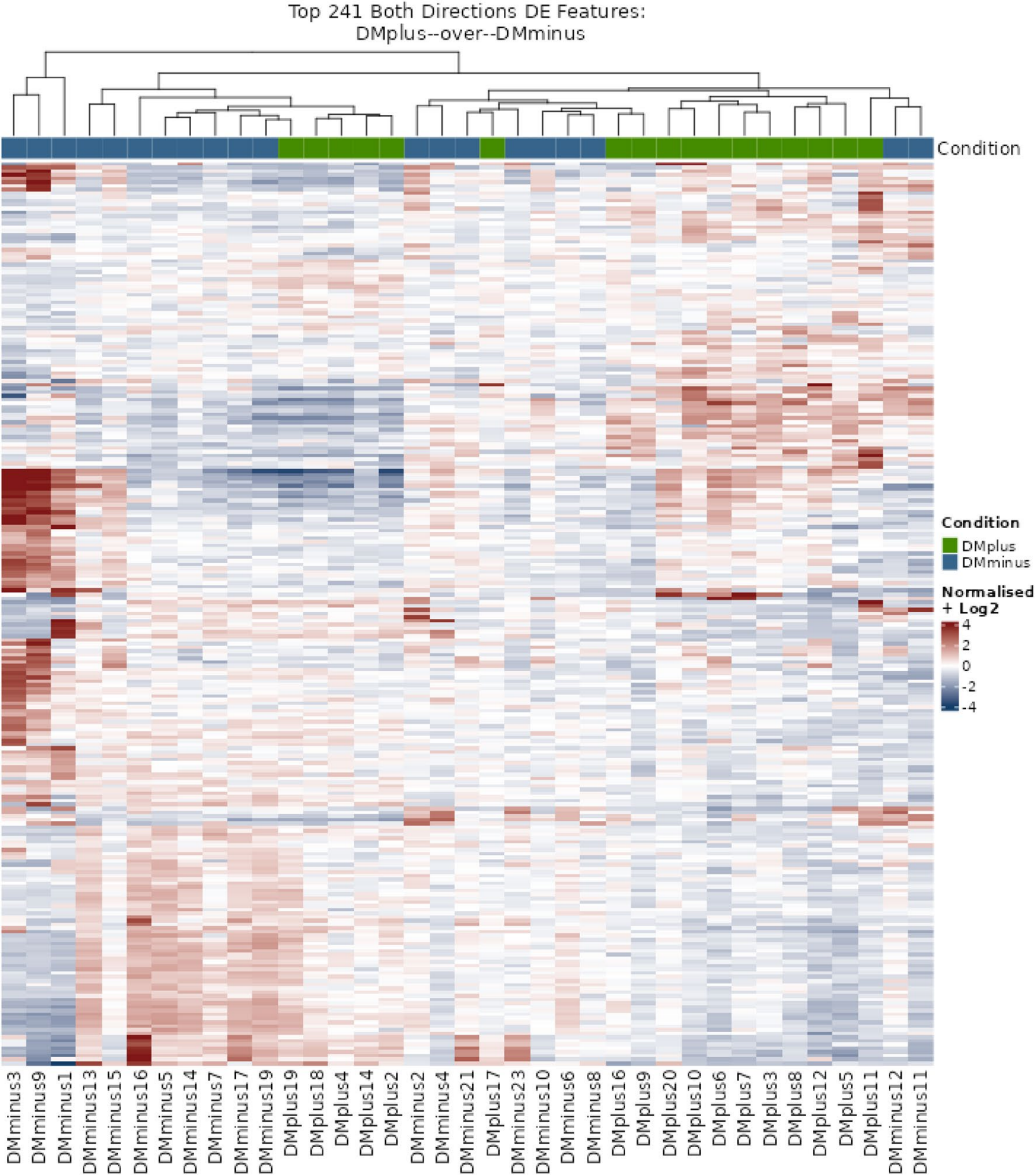
Heatmap of the most differentially expressed lncRNAs in the whole tissue of PAD patients with (DMplus) and without (DMminus) diabetes mellitus (rows indicate the expression of 241 most expressed lncRNAs, columns indicate the individual samples). Clustering was performed using conditional formatting features. Even if many samples are clustered, high heterogeneity was observed regarding the study groups.

The heatmap of the most differentially expressed lncRNAs in the tissue of PAD patients with DM (DMplus) and without DM (DMminus) revealed broad heterogeneity of the individual samples regarding the expression of lncRNAs (Fig. 3). Even if many samples were clustered, each study group was distributed throughout the whole heatmap.

### Differential expression analysis of lncRNA in SMCs of the PAD tissue samples

**Figure 4:**
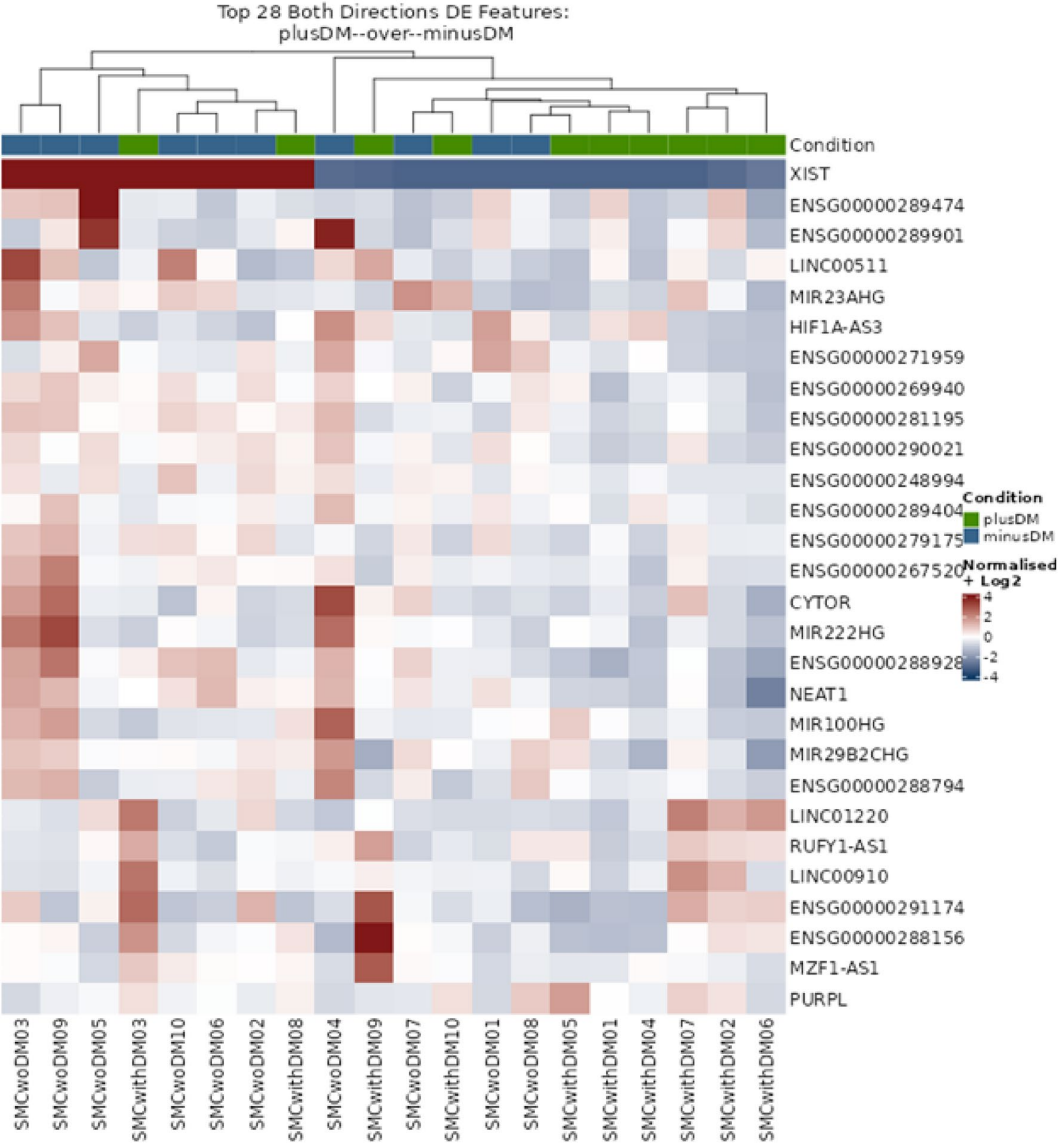
Heatmap of the most differentially expressed lncRNAs in the SMCs from PAD patients with (DMplus) and without (DMminus) diabetes mellitus (rows indicate the expression of 28 most expressed lncRNAs, columns indicate the individual samples). Clustering was performed using conditional formatting features.

Regardless of the preselection of the study samples according to their histology, wide heterogeneity of the lncRNA expression in the whole tissue was still observed. Therefore, we changed our experimental approach and focused only on the SMCs within the study samples. Consequently, we performed a dissection of the tissue and analysed the lncRNAs only from the microdissected SMC areas. The corresponding volcano plot is shown in Fig. 2b, and the heatmap of the clustering of the differentially expressed lncRNAs is shown in Fig. 4. The expression of lncRNA only from SMCs showed markedly higher homogeneity and better distribution (clustering) of the study samples (Fig. 4) compared to the heatmap of the whole tissue (Fig. 3).

**Figure 5:**
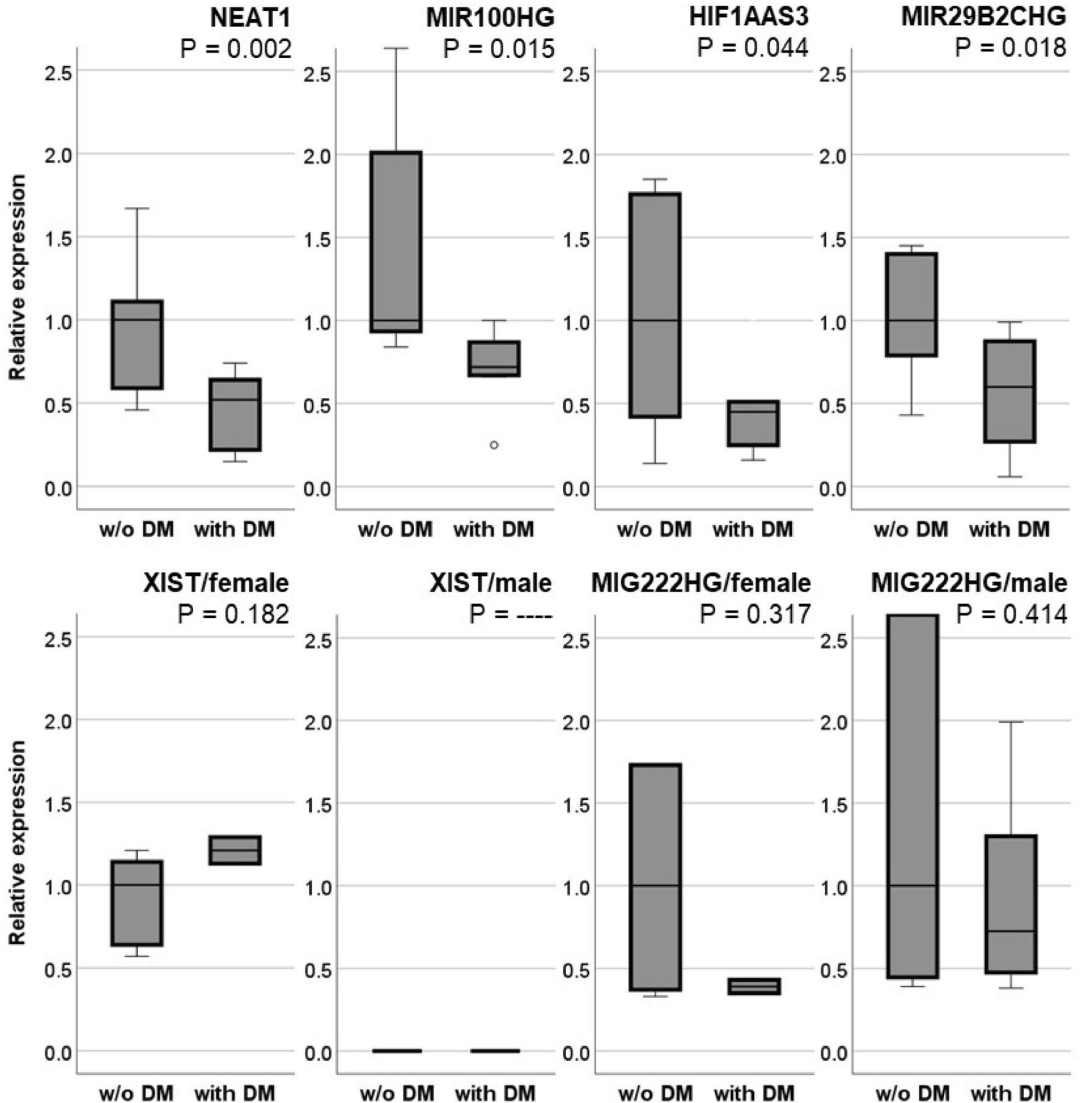
Box plots using RT-PCR to compare the expression of relevant lncRNAs in SMCs between the study groups (with and without DM). The lower part shows subsequent analysis of XIST and MIG222HG separated for sex because these two lncRNAs are expressed only on chromosome X. The expression was normalised for the housekeeping gene GAPDH. Furthermore, for better comparison, the expression of the individual lncRNAs of the study group without DM (w/o DM) was set as references equal 1.

In total, 14,499 lncRNAs could be detected, with 761 lncRNAs with counts above the threshold of 10 counts per million. From these 761 lncRNAs, 28 demonstrated significantly differential expression between the study groups (FDR < 0.1) (Table 3). Of these 28 lncRNAs, 14 were novel transcripts not yet described. The function of two other lncRNAs, MIR23AHG and RUFY1-AS1, is also unknown (Table 3). Six of the remaining lncRNAs have been described in different types of cancer (*LINC01220, CYTOR, LINC00910, LINC00511, PURPL, and MZF1-AS1*), one with macrophage polarisation (*MIR222HG*), and five of these lncRNAs could be associated with cardiovascular disorders (CVDs), mainly with SMC proliferation, migration, apoptosis, and phenotypic switch (*NEAT1, XIST, MIR100HG, HIF1A-AS3, and MIR29B2CHG*) (Table 3). The latter lncRNAs were further investigated and RT-PCR was performed to confirm the results from the transcriptome analysis (Fig. 5).

RT-PCR analysis of NEAT1, MIR100HG, HIF1A-AS2, and MIR29B2CHG confirmed the results from the RNA sequencing with a significant reduction of their expression in SMCs of PAD patients suffering from DM (Fig. 5). XIST and MR222HG were expressed only on chromosome X. Therefore, additional subsequent adjustment for sex was performed. Interestingly, the lncRNA XIST was expressed only in females and after the correction for sex, no significant differences were observed (Fig. 5). The same results were found for MIR222HG, even if, in this case, the expression was found in males and females (Fig. 5).

In addition, due to the rather low amount of samples in each study group, we performed a post-hoc power analysis. For NEAT1 (98.1%), HIF1A-AS3 (82.3%), and MIR29B2CHG (83.7%), the statistical power was > 80%. In contrast, MIR100HG showed values < 80% (61.2%). Furthermore, to elucidate a potential clinical significance, receiver operating characteristic (ROC) curve analysis for the significantly differentially expressed lncRNA was performed (Supplementary Fig. 1). For the lncRNAs NEAT1, HIF1AAS3, and MIR29B2CHF, the AUC was significant (*P* = 0.001, 0.007, and 0.011). For MIR100HG, the AUC was not statistically significant (*P* = 0.290).

### Gene enrichment analysis

**Figure 6:**
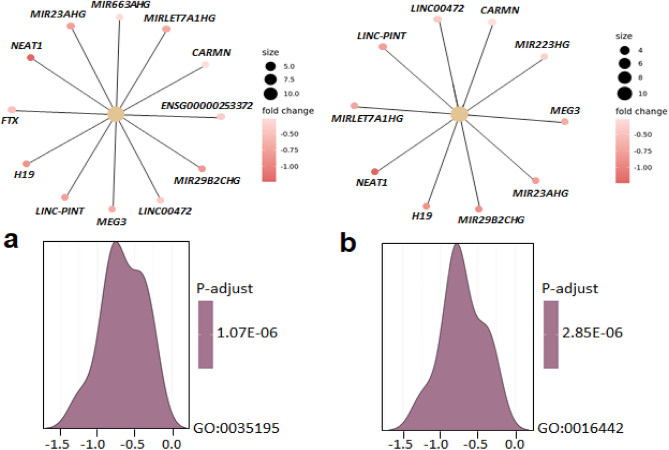
Gene set enrichment analysis (GSEA). (**a**) Biological process (BP): GO:0035195—miRNA-mediated post-transcriptional gene silencing pathway. (**b**) Cellular component (CC): GO:0016442—RISC complex pathway. The corresponding enrichment data are summarised in Table 2.

In order to identify groups of lncRNA genes that are over-represented in our experimental setup, we performed, in addition, a gene and pathway enrichment analysis with the 761 lncRNAs having counts above the threshold (Fig. 6, Table 4). The gene enrichment pathway analysis (GEPA) revealed the involvement of insulin-like growth factor 2 mRNA binding proteins and diabetes pathways associated with the lncRNA H19. However, only the first pathway showed statistical significance (P-adjusted < 0.1) (Table 4). The gene set enrichment analysis (GSEA) showed significant enrichment for genes involved in miRNA-mediated post-transcriptional gene silencing (GO:0035195), including the RISC complex (GO:0016442) (Fig. 6, Table 4).

**Table 4:** Gene enrichment pathway analysis (GEPA) and gene set enrichment analysis (GSEA).

	*P* value	*P*-adjusted	Odds Ratio	Combined Score	Genes	
Binding of RNA by insulin-like growth factor 2 mRNA binding proteins (IGF2BPs/IMPs/VICKZs)	0.0143	0.0572	81.46	345.92	*H19*	
Diabetes pathways	0.2194	0.4388	4.16	6.32	*H19*	

ID	Description	Size	NES	*P* value	*P*-adjusted	geneName
BP GO:0035195	miRNA-mediated post-transcriptional gene silencing	14	− 1.942	5.3E−07	1.1E−06	CARMN/MIR663AHG/ ENSG00000253372/ LINC00472/FTX/MEG3/ MIRLET7A1HG/ MIR23AHG/ LINC-PINT/H19/ MIR29B2CHG/NEAT1
CC GO:0016442	RISC complex	11	− 1.863	1.4E−06	2.8E−06	

The silencing complex included, besides the already significantly different lncRNAs (DMplus vs DMminus) such as NEAT1 and MIR29B2CHG, also other relevant lncRNAs involved in SMCs physiology or CVDs, such as CARMN, FTX, and MEG3.

The enrichment score was calculated for each annotation category by screening all the genes from a differential expression analysis and their associated fold changes. GO—gene enrichment; Sub-ontologies of GO hierarchy: BP—biological process, CC—cellular components; NES—normalised enrichment score. The corresponding graphical illustration is shown in Fig. 6.

## Discussion

The current study identified a couple of lncRNAs significantly downregulated in SMCs of PAD individuals suffering from DM, which might be of interest for further investigation to elucidate their role in diabetes. Sixteen of them (e.g. ENSG00000289474, ENSG00000288156 or ENSG00000289901) are so far of unknown function. Others, such as LINC01220, CYTOR, LINC00910, LINC00511, PURPL, and MZF1-AS1, have been described so far only in cancer. Four differentially expressed lncRNAs have already been associated with CVDs, mainly with SMC function and phenotypic switch (NEAT1, MIR100HG, HIF1A-AS3, and MRI29B2CHG). These lncRNAs might serve as potential diagnostic markers or therapeutic targets to improve the function of SMCs within the diseased tissue.

Comparing the expression pattern of lncRNAs in the whole tissue of PAD patients with and without DM, only ACTA2-AS1 showed significantly increased expression in the DMplus study group. The inability to detect other differentially expressed lncRNAs in the tissue is mainly due to the broad heterogeneity in the underlying pathomorphology, even after our careful preselection. All samples were highly atherosclerotic, and the additional burden of DM was, therefore, not easy to distinguish. The significantly differentially expressed lncRNA ACTA2-AS1 has been described so far, mainly in the context of cancer development^26–28^. However, Arencibia et al. found that the ACTA2-AS1 might also regulate the phenotypic switch and proliferation of SMCs^29^. In addition, the downregulation of ACTA2-AS1 shows significant enrichment in collagen biosynthesis and alternation in the structure and composition of the extracellular matrix (ECM). Consequently, ACTA2-AS1 might also play an essential role in PAD patients with DM by affecting the function of SMCs.

Due to the above-mentioned lack of differentially expressed lncRNAs in the whole tissue samples caused by the extended atherosclerotic pathology and wide heterogeneity, we focused on the SMCs within the concerned specimens. In this experimental approach, 28 significantly differential expressed lncRNAs were detected between the study groups. Fourteen of these lncRNAs were novel transcripts. Some of them, for instance, ENSG00000289474, with a 12-fold reduction in its expression in PAD patients with DM, might be of interest to be further investigated. Many of the remaining lncRNAs, such as LINC01220, CYTOR, LINC00910, LINC00511, PURPL, and MZF1-AS1, have been described in association with different types of cancer^30–35^. Interestingly, the expression changes were associated mainly with cell proliferation and migration of the tumour cells. So far, there is no study examining these lncRNAs in CVDs. Consequently, whether these lncRNAs also affect SMCs has not yet been investigated. MIR222HG was also described in association with cancer^36,37^ but also with macrophage polarisation^38^. In this context, it is to be noted that SMCs can adopt various phenotypes, including also that of macrophages or foam cells^39^. Thus, lncRNA MIR222HG might also be involved in the phenotypic switch of SMCs, for example, resembling the macrophage phenotype and taking up lipids and cholesterol derivatives. Various lncRNAs have been described to regulate SMCs plasticity^40^.

Four significantly differentially expressed lncRNAs between our study groups with and without DM, namely *NEAT1, MIR100HG, HIF1A-AS3,* and *MIR29B2CHG*, have already been associated with CVDs^32,41–48^, mainly with the proliferation, migration, apoptosis, and phenotypic switch of SMCs (Table 3)^41,46,47,49–51^.

LncRNA *NEAT1* (nuclear-enriched abundant transcript 1) is involved not only in atherosclerosis^44,46^ or aneurysm^52^ but has shortly been mentioned as being also dysregulated in DM, especially in diabetic neuropathy and diabetic kidney disease^53–56^. Furthermore, *NEAT1* has already been described as having a critical in the phenotypic switching of SMCs by repressing SM-contractile gene expression through an epigenetic regulatory mechanism^16^. In addition, overexpression of *NEAT1* participates in proliferation and can inhibit apoptosis of SMCs^41^, improving their ability to synthesise ECM components in order to stabilise the arterial wall. Thus, the reduction of *NEAT1* expression in PAD patients with DM might further impair the function of SMCs and aggravate the clinical outcome.

Regarding lncRNA *HIF1A-AS* (hypoxia-inducible factor-1 alpha antisense RNA), only *HIF1A-AS1* and *-AS2* have been described to modulate the proliferation, migration, and phenotypic switch in SMCs, particularly in aortic aneurysm^47,50,59,60^. So far, there is no information about the role of *HIF1A-AS3* in CVDs. However, because the other isoforms affect the behaviour of SMCs, it can be assumed that *HIF1A-AS3*, significantly downregulated in the DMplus study group, might also affect the function of SMCs. The lncRNAs *MIR100HG* and *MIR29B2CHG* have only been mentioned in the context of cardiomyopathy and heart failure^42,48^. So far, there is no information about their potential role in PAD, DM or SMCs. Whether or how far these two lncRNAs might be involved in the corresponding pathophysiology is so far unknown and has to be further investigated.

Interestingly, considering the gene enrichment pathway analysis, additional lncRNAs occurred to play an essential role in the pathology of DM in PAD patients. Of particular interest were *H19, CARMN,* and *MEG3.* Our group and others have already described the critical role of lncRNA *H19* in atherosclerosis and aneurysm^61–63^. Furthermore, *H19* has long been proven to be connected with the phenotypic switch of SMCs^64^ and diabetes, especially in the regulation of glucose metabolism^65,66^. In our experimental setup, *H19* was involved in the binding of RNA by insulin-like growth factor 2 mRNA binding proteins (IGF2BPs). IGF2BPs bind specific sets of RNAs, regulating their translation, stability, and subcellular localisation^67^. IGF2BPs, belonging to the IGF mRNA binding proteins (IMPs), are highly conserved and believed to play an essential role in cell migration, metabolism, stem cell renewal, and development^68^. In adults, their expression is either absent or at very low levels in most tissues. However, IMPs, particularly IGF2BPs, appear to resume their physiological functions in tumour cells and exhibit multiple attachments to *H19* lncRNA^69^. Altogether, mRNA binding protein family IMPs/IGF2BPs has been described in a plethora of biological processes, including development, tumorigenesis, and differentiation^68^. Furthermore, in diabetic patients, IMP genes have been associated with impaired insulin secretion^68^. Whether and how far vascular cells, particularly SMCs, can be affected has not yet been investigated.

Regarding the gene set enrichment analysis, *H19* seemed to be involved again, together with *NEAT1, CARMN, MIR23AHG, MIR29B2CHG, MIR663AHG, MIRLET7A1HG, LINC-PINT, FTX*, and *MEG3*. Of these lncRNAs, irrespective of the already described *NEAT1, MIR29B2CHG*, and *H19*, the lncRNA *CARMN* and *MEG3* have already been described in the context of CVDs and SMCs^40,66,70–73^. The lncRNA *CARMN* has been associated with atherosclerosis^40,72^ and SMC plasticity^70^. Dong et al. observed that *CARMN* overexpression maintains SMCs in the contractile phenotype^70^. On the contrary, loss of CARMN, as observed in our study, affects the phenotypic plasticity of SMCs and significantly accelerates atherosclerosis^40,71^. The lncRNA *MEG3* has already been considered an essential lncRNA in CVDs and modulation of SMC phenotype^64,73^. Recently, *MEG3*, among other lncRNAs such as *MIAT*, *H19*, *MALAT1*, *AVRIL*, and *HOTAIR*, has been connected with several diabetic complications, being either upregulated or downregulated depending on the disease context or regulatory partners^43,66,74^. These lncRNAs are involved particularly in oxidative stress, inflammation, apoptosis, and angiogenesis pathways, mediating either protective function or contributing to the severity of DM^66^.

## Limitations

The limitation of this study is the relatively small number of tissue samples and the broad heterogeneity between the individual specimens. Furthermore, it should be noted that the atherosclerotic patterns in PAD patients with and without DM are usually different, and thus, the results may have also been affected by the sampling site. In order to increase the homogeneity within the study groups, extended histological characterisation of the samples was performed, and tissues with great extent of calcification, inflammation and low amount of cells, which markedly differed from the average, were excluded. In this manner, from the 79 characterised specimens, only 37 were involved in the study. The semi-quantitative analysis of the included samples showed no significant differences between the specimens afterwards (see Table 2). Nevertheless, a more extensive or consecutive study with other larger tissue sample sets is necessary to confirm the results of our present work.

## Conclusion

In our current study, we have analysed for the first time lncRNAs in PAD patients suffering from DM, using extended transcriptomics analysis of the corresponding tissue and the underlying SMCs. Particularly in SMCs, 28 significantly differential expressed lncRNAs were detected. Sixteen of them were novel transcripts or so far with an unknown function. Some of them, such as for instance *ENSG00000289474* with a 12-fold expression reduction in PAD patients with DM, might be of interest to further investigation. Many of the other lncRNAs, such as *LINC01220, CYTOR, LINC00910, LINC00511, PURPL, and MZF1-AS1,* have been described in association with different types of cancer, mainly with proliferation and migration of the tumour cells. These lncRNAs have not yet been analysed in the context of CVDs and might affect the pathophysiological behaviour of vascular cells within the diseased tissue. Five of the differentially expressed lncRNAs, namely *NEAT1, HIF1A-AS3, MIR100HG, and MIR29B2CHG,* have already been proven to play a role in CVDs, especially in proliferation, migration, apoptosis, and phenotypic switch of SMCs. Interestingly, all these lncRNAs were downregulated in PAD patients with DM, having a negative effect on SMCs, thus being able to aggravate the clinical outcome. Furthermore, enrichment analysis revealed additional lncRNAs, such as *H19*, *CARMN*, and *MEG3*, with a possible role in DM and the fate of SMCs. In summary, our study detected a set of relevant lncRNAs affecting the SMCs in the PAD patients suffering from DM, which might serve as potential therapeutic targets to improve the function of SMCs within the diseased tissue and, thus, the clinical outcome for these patients.

## Material and methods

All methods were performed in accordance with the corresponding relevant guidelines and regulations.

### Tissue samples

Vascular tissue samples of the iliac artery were obtained from patients suffering from peripheral arterial disease (PAD) with and without diabetes mellitus (DM) (n = 79) who underwent open surgical intervention in our Department of Vascular Surgery (USZ/UZH), collected in our Swiss Vascular Biobank (SVB). All patients gave appropriate written informed consent. The local ethics committee (Cantonal Ethics Committee Zurich, Switzerland; BASEC-No 2020-00378 and 2020-01844) approved the tissue sample collection and analysis procedure. All tissue samples were divided for consecutive histological and molecular-biological analyses. For histological studies, the tissue was fixed in 4% formalin and embedded in paraffin (FFPE). Adjacent pieces of the corresponding specimens were immediately frozen and stored at − 80 °C.

### Histological evaluation

For a pathomorphological evaluation of the PAD tissue (n = 79), sections of FFPE samples were stained with Haematoxylin–eosin (HE) and Elastica van Gieson (EvG), focusing on cellular composition, inflammatory infiltration, calcification, and distribution of the overall content of collagen and elastin. For immunohistochemistry (IHC), FFPE sections were mounted on pre-coated (0.1% poly-L-lysine; Merck/Sigma Aldrich, Buchs, Switzerland) SuperFrost Plus slides (ThermoFisher Scientific), and antigen retrieval was performed by heat in citrate buffer (pH 6.0). Primary antibodies used in this study were purchased from Lucerna-Chem (Luzern, Switzerland) and Agilent/Dako (Basel, Switzerland): alpha-SM-actin for SMCs (abcam ab5694, 1:100), CD31 for ECs (ab134168, 1:40), CD45 for leukocytes (M0701, 1:2,000), CD68 for macrophages (M0814, 1:2,000), MYH10 for synthetic SMCs (ab230823, 1:200), and MYH11 (ab224804, 1:400) were diluted in DAKO REAL Antibody Diluent (Agilent/Dako). To detect the primary antibody, mouse/rabbit specific HRP/DAB (ABC) detection IHC kit (ab64264, abcam) and Mayer’s haematoxylin (Carl Roth, Switzerland) for the nuclear counterstain was used. All slides were digitalised by Zeiss Axio Scan.Z1 and Zeiss Zen Lite 2.1 software.

### RNA extraction and quality determination

For the transcriptome analysis of the whole samples, RNA was isolated from fresh-frozen tissue using the TRIZOL reagent (ThermoFisher), which enables the extraction of total RNA, including non-coding RNAs. The tissue samples were homogenised in a volume of 1 mL TRIZOL, mixed with 200 µL chloroform, and centrifuged. The upper (clear) phase containing the RNA was carefully transferred into a new tube, precipitated with isopropanol, washed with 75% ethanol, the RNA pellet air-dried, suspended in 50 µL RNase-free water and stored at − 80 °C until further use.

For the transcriptome analysis of SMCs, FFPE tissue was used in order to be able to perform reliable manual microdissection and to remove only areas containing the cells of interest. Briefly, all samples were stained consecutively with alpha-SM-actin to detect SMCs. Next, a consecutive slide of 25 µm thickness without staining was prepared, and corresponding areas positive for SMCs were excised under the light microscope (Motic AE200) with sterile scalpels. Six to eight slices from the same samples were pooled, and RNA was isolated using the High Pure RNA Paraffin Kit (Roche, Switzerland) according to the manufacturer’s protocol.

The RNA concentration was determined by NanoDrop Lite Plus Spectrophotometer (Witec, Sursee, Switzerland). To evaluate the RNA quality and the degree of degradation in order to select suitable samples for RNA sequencing (atherosclerotic tissue samples have markedly lower RNA quality than healthy tissue^75^), the RNA integrity numbers (RINs) and DV200 index (percentage of fragments of > 200 nucleotides) were determined (TapeStation 4150; Agilent)) using standard Agilent RNA ScreenTape Assay or Agilent High Sensitivity RNA ScreenTape Assay depending on the RNA concentration in accordance with the manufacturer’s protocols. All measurements were performed in duplicates, and the experiments were repeated in cases of high variability or discrepancies.

### Library preparation, RNA sequencing, and RT-PCR analysis

The library preparation and RNA sequencing were performed in collaboration with the Functional Genomic Center Zurich (FGCZ/ETH, Switzerland). In order to be able to analyse non-coding RNAs, the ribosomal RNA depletion method was applied. Briefly, the RNA library was prepared using the SMARTer Stranded Total RNA-Seq Kit from Clontech/Takara Bio (USA) according to the manufacturer’s protocol. The fragmented samples were reversed transcribed into cDNA, end-repaired, polyadenylated, and TruSeq adapters containing the index for multiplexing were ligated at the ends of each fragment. Fragments containing the TruSeq adapters were selectively enriched by PCR. The quality and quantity of the RNA library were validated again by the determination of RIN. The RNA sequencing was performed on the Illumina NovaSeq 6000 (Illumina, Berlin, Germany) with 200 M reads in 100 cycles.

In order to validate the results for the significantly differentially expressed lncRNAs associated with CVD (NEAT1, MIR100HG, MIR29B2CHG, and HIF1A-AS3), additional quantitative real-time PCR (RT-PCR) was performed using TaqMan approach and master kit (ThermoFisher Scientific) on QuantStudio 5.0 (Agilent). The cDNA was synthesised with the RevertAid First Strand cDNA Synthesis Kit (ThermoFisher Scientific). The expression of all lncRNAs was normalised for the housekeeping gene glycerinaldehyde-3-phosphate dehydrogenase (GAPDH).

### Data analysis

The data analysis was performed with the SUSHI framework^76,77^, developed by the FGCZ ETH/UZH, and using the open Galaxy platform. Spliced Transcripts Alignment to a Reference (STAR) software was used to align the human RNA-Seq dataset^78^. The quality control of the individual reads was proven by FastQC. The read alignment was performed by STAR, and the estimation of the reads abundance by FeatureCounts from the R package subreads^79^. A minimum of 10 reads in at least one group of replicates was required to consider a gene as detected. Differential expression analysis was assessed using the linear model approach from the Bioconductor package DESeq2 and EdgeR2^80^. The correction for multiple testing was obtained with the Benjamini–Hochberg algorithm calculating the False Discovery Rate (FDR, adjusted *P*-value)^81^. The thresholds of FDR < 0.1 was considered significant. All other statistical analyses were performed using IBM’s SPSS software version 29 (SPSS Inc., Chicago, IL, USA). An independent t-test and a Levene test of equality of variances were used. The statistical analyses were two-sided, with *P* < 0.05 as the significance level.

## Data availability

All datasets generated and analysed during the study are stored at the servers of Functional Genomic Server Zurich (FGCZ) (Eidgenoessiche Technische Hochschule Zuerich/Switzerland) (project ID 28383). Datasets used in the current publication are stored at http://www.ncbi.nlm.nih.gov/bioproject/1072644.

### Supplementary Information

Supplementary Figure 1.
