# Supplementary material for: Transcriptomics analysis of long non-coding RNAs in smooth muscle cells from patients with peripheral artery disease and diabetes mellitus
Source: Sci Rep. 2024 Apr 14;14:8615. doi: 10.1038/s41598-024-59164-7 (PMC11016542; doi:10.1038/s41598-024-59164-7)
Supplement: Supplementary file 1 — Supplementary Figure 1. [file 41598_2024_59164_MOESM1_ESM.pptx]

## Slide 1
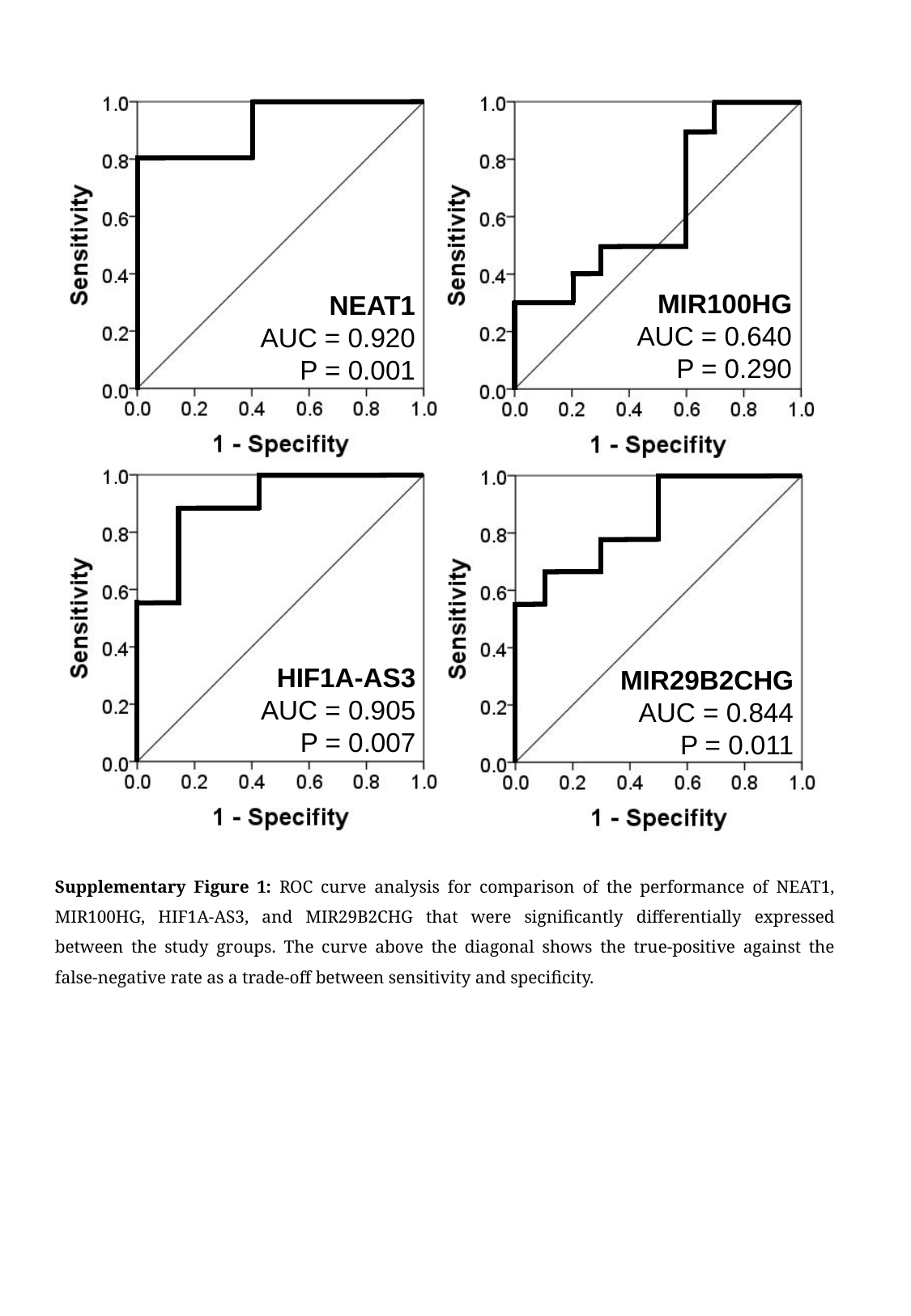

NEAT1
AUC = 0.920
 P = 0.001
MIR100HG
AUC = 0.640
 P = 0.290
HIF1A-AS3
AUC = 0.905 P = 0.007
MIR29B2CHG
AUC = 0.844
 P = 0.011
Supplementary Figure 1: ROC curve analysis for comparison of the performance of NEAT1, MIR100HG, HIF1A-AS3, and MIR29B2CHG that were significantly differentially expressed between the study groups. The curve above the diagonal shows the true-positive against the false-negative rate as a trade-off between sensitivity and specificity.
